# Supplementary material for: Assessment of regional body composition, physical function and sarcopenia among peruvian women aging with HIV: A cross-sectional study
Source: PLOS Glob Public Health. 2023 Aug 18;3(8):e0000814. doi: 10.1371/journal.pgph.0000814 (PMC10437949; doi:10.1371/journal.pgph.0000814)
Supplement: Attachment — Submitted filename: Letter of Response_4.27.23_F.docx [file pgph.0000814.s001.docx]

April 27, 2023

Catherine Kyobutungi

Madhukar Pai

Editors-in-Chief

PLOS Global Public Health

Dear Editors-in-Chief:

We appreciate the thoughtful comments and suggestions from the reviewers and editorial team and would appreciate if our manuscript could be considered for publication on PLOS Global Public Health. Please see below the responses for each reviewer’s comments and journal requirements.

**Journal Requirements**

1. **Please send a completed 'Competing Interests' statement, including any COIs declared by your co-authors. If you have no competing interests to declare, please state "The authors have declared that no competing interests exist". Otherwise please declare all competing interests beginning with the statement "I have read the journal's policy and the authors of this manuscript have the following competing interests:"**

The statement regarding the competing interests has been updated.

2. **Please amend your detailed Financial Disclosure statement. This is published with the article. It must therefore be completed in full sentences and contain the exact wording you wish to be published.**

The financial disclosure statement has been updated.

3. **State what role the funders took in the study. If the funders had no role in your study, please state: “The funders had no role in study design, data collection and analysis, decision to publish, or preparation of the manuscript.” If any authors received a salary from any of your funders, please state which authors and which funders.**

The statement has been updated under “Funding” (page 27)

4. **Please indicate by return email the full and correct funding information for your study and confirm the order in which funding contributions should appear.**

We will send the correct funding information with funding contributions via email. They also appear on the last page under financial disclosures in pages 26-27.

**Reviewers Comments**

**Reviewer #1: I think it is crucial to specify in the introduction that HIV infection became a chronic condition because of the antiretroviral treatment. In the methodology, the authors have to indicate if the status of being on antiretroviral treatment is an inclusion criterion and was the minimum duration on it for the inclusion in the study. It could be a good point to specify if they have tried to match women of the two group regarding the age groups. Finally, I deplore the fact that no women were on anti-integrases since it is a recent class of drugs although the cross-sectional character of the study would not have allowed the establishment of a causal link.**

*Response to Reviewer #1:* We appreciate your comments and suggestions for this manuscript. We have now specified in the introduction that HIV infection has become a chronic infection due to effective antiretroviral treatment (Line 56-58, page 3). In the methodology, we have also updated that all participants in the group of women living with HIV were on antiretroviral treatment (line 106-108, page 4), but there was no minimum treatment duration required as part of our inclusion criteria. We did not match women in the control group 1:1, preferring to adjust for differences in covariates in the multivariable analyses. We did however recruit women who were also ≥40 years, and from the same surrounding community. Finally, we agree with the reviewer regarding the importance of discussing the impact of different antiretroviral agents/classes on skeletal outcomes and have now added additional detail in the RESULTS (lines 204-212) regarding this. It is important to note that during the time of this study, in our population of patients living with HIV, almost all patients were treated with one of the free combination ART regimens sponsored by the National HIV/STI Program. The endorsed first-line regimen consisted of TDF, 3TC or FTC, and EFV. Second-line regimens included TDF-, ABC- or AZT-based, with one NNRTI plus LPV/r or DTG, or two NNRTIs. As a very small number of patients were on integrase inhibitors at the time our sample was enrolled, we were not able to analyze the association between this agent and our outcomes of interest. We agree that it will be important for future larger prospective studies to assess the relationship between different antiretroviral classes and our outcomes of interest.

**Reviewer #2: This paper presents an original study that aims to analyze the relationship between HIV and body composition changes, physical function and sarcopenia among women from Latin America and Caribbean. This is an interesting study which provides important results on the health impact of chronic HIV. The strengths of this study are: (1) it includes a control group and (2) it uses Dual X-ray absorptiometry (DXA) to assess body composition. On the other hand, the sample size was small with limited power for the analysis and the evaluation of physical function was limited to the SPPB test. Some aspects of the analysis need also to be clarified before the paper could be published. My main comments are listed below.**

**ABSTRACT
Major comment
The authors should be more careful on their statement about a greater prevalence of severe sarcopenia among HIV+ as it is not significant (only 4 and 2 cases with a p value of 0.8).
Minor comment
The authors may include the study design in the paper’s title.**

*Response to Reviewer #2*: We agree with the reviewer’s comments regarding the abstract and have now clarified both with numbers and text that the increased percentage of severe sarcopenia among women with HIV did not reach statistical significance. In terms of the title, we have now added the study design to the paper title.

**Reviewer #2: INTRODUCTION
The authors state that the “musculoskeletal system is significantly affected by HIV and its treatment”, and later explain that this is related to the “decreased bone mineral density, bone mass and [to] heterogeneous disorder of body composition” occurring in HIV-infected people. This section may benefit of being more developed. For instance, why HIV and its treatment result in these negative outcomes and how are they related,**

*Response to Reviewer #2:* We appreciate this suggestion; we have expanded upon our explanation on lines 72-74, page 3.

**Reviewer #2: Line 70. What do the authors mean by “the heterogeneity of PLWH”. Do they refer to the heterogeneity of the situations?**

*Response to Reviewer #2:* By heterogeneity of older PLWH we refer to both sociodemographic and clinical characteristics that may differ among individuals with HIV.

**Reviewer #2: It is not clear if body composition changes include sarcopenia as suggested line 67 or not (as suggested line 81).**

*Response to Reviewer #2:* Thank you for this comment. We have now updated the sentence in line 74 to say *“Alterations in body composition, such as changes in muscle mass and fat redistribution, can reflect important changes in bone and MSK health [7]. Changes in muscle mass, together with declines in muscle performance/function and strength, which can be seen with aging, contribute to development of sarcopenia, which has been independently associated with morbidity and mortality [8].”*

**Reviewer #2: The objectives presented lines 83 - 84 would benefit to be more specific:
Which relation will be analyzed? What are the assumptions to be verified or tested? Or is it a fully exploratory study (which seems not the be the case from the background section).**

*Response to Reviewer #2:* We appreciate these comments. We have expanded our description of our study objectives in lines 93-98, page 4 to say *“We aimed to measure physical function, physical strength and sarcopenia among middle-aged and older Peruvian women with and without HIV using well-established rigorous measures, and to explore the association of the outcomes in the two groups with key clinical and sociodemographic risk factors. We hypothesized that in addition to age, certain HIV-associated factors (e.g., CD4+ count, duration of HIV, exposure NRTIs or PIs) would be associated with outcomes among women with HIV in our study.*”

**Reviewer #2: METHODS
The presentation of the study design, setting and population as well as of the variables used and the measurement methods is clear and detailed enough. On the other hand, the statistical section could be improved.
First, the authors may explain how the study sample size was arrived at. Moreover, the authors used linear regression but did not explain if the normality of the variables was checked and how non-normal distribution was managed. They may also explain if there were missing values and how they were managed.**

*Response to Reviewer #2:* We appreciate these comments regarding the methodology of this manuscript. Regarding our sample size, this study was the secondary aim of a larger study which focused on vertebral fracture and osteoporosis outcomes (Aim 1 and primary outcomes of interest) and health-related quality of life measures (Aim 3 of the study). Our study was powered for the primary outcome of interest, namely vertebral fractures, which occur with relatively low prevalence. Specifically, we originally aimed to enroll a sample of 244 women per group with an α = 0.05, and 80% power to detect a significant difference in fracture prevalence between the two study groups. Of note, our final sample did not quite achieve the planned targets as we ended recruitment early (March 2020) due to the COVID-19 pandemic, and a portion of patients recruited in both groups were not able to complete the imaging portion of the study.

However, our final numbers per group still yielded 80% power for this sub study based upon our original parameters. If we calculate a sample size calculation using some of our main outcomes such as Grip Strength, based on the paper by Crane et al 2019 (3) looking for physical function among patients aging with HIV vs HIV-uninfected: feasibility of using the SPPB in clinical care of PLWH aged 50 or older, she found a mean grip strength of 26.5 among women with HIV compared to 23.4 among women without HIV, with these two means we would need a sample size of 78 per group (HIV-infected and uninfected) to have a power of 80% with a confidence interval of 95% which we have achieved. Moreover, if we calculate a sample size calculation using another of our main outcomes such as Sarcopenia spectrum, based on the paper by Pinto Neto, et al 2016 (4) looking for HIV and its association with sarcopenia, albeit using somewhat different measurement tools than our study, she found a frequency of 33% of sarcopenia spectrum (presarcopenia and sarcopenia) among people with HIV, compared to a frequency of 13% among people living without HIV, with these two frequencies we would need a sample size of 37 among people with HIV and 73 among people without HIV to have a power of 80% with a confidence interval of 95% which we have achieved.

Regarding the regression models, thank you for asking for clarification, we have now updated the manuscript accordingly in Statistical analysis starting in Line 166, page 6. As part of the analyses, we tested covariates for multicollinearity and ensured our data fulfilled the assumptions required to utilize linear regression analysis prior to performing the analyses. Most of our data followed a normal distribution, that one that did not were the nadir viral load and current viral load but those were not used for comparisons matter. Finally, for all the participants presented in this study, we did not have any missing values for the covariates studied as we prepared a detailed SOP for study procedures in advance and followed it carefully during data collection. This included double checking data entry daily and weekly to assure there were no missing values.

References:

1. Ilha TASH, Comim FV, Copes RM, Compston JE, Premaor MO. HIV and Vertebral Fractures: a Systematic Review and Metanalysis. Sci Rep. 2018 Dec;8(1):7838.
2. Clark P, Cons-Molina F, Deleze M, Ragi S, Haddock L, Zanchetta JR, et al. The prevalence of radiographic vertebral fractures in Latin American countries: the Latin American Vertebral Osteoporosis Study (LAVOS). Osteoporos Int. 2009 Feb;20(2):275–82.
3. Crane H, MIller M, Pierce J, et al. Physical Functioning Among Patients Aging With Human Immunodeficiency Virus (HIV) Versus HIV Uninfected: Feasibility of Using the Short Physical Performance Battery in Clinical Care of People Living With HIV Aged 50 or Older. 2018. <https://www.ncbi.nlm.nih.gov/pmc/articles/PMC6411210/>
4. Pinto Neto L, Cerqueria M, et al. Braz J Infect Dis. 2016 Jan-Feb; 20(1): 99–102. doi: 10.1016/j.bjid.2015.10.003. https://www.ncbi.nlm.nih.gov/pmc/articles/PMC9425396/

**Reviewer #2: RESULTS**
**The paper may benefit of more information on the study flow. For instance, how many participants refused to participate (and what were their characteristics).
Table 2: the difference in the proportion of HIV+ and HIV- participants with SPPB ≤8 should be tested only once overall (and not 2 p-values). P-value for the Chi2 test cannot be 0.09 or <0.0001**

*Response to Reviewer #2:* We appreciate your input regarding the results section of this manuscript. In terms of the study flow, we agree that it would be quite helpful to have more information regarding the different stages in study recruitment, including the number of patients who declined to participate. We unfortunately did not collect this data as referring physicians were not asked to record information regarding patients who were not referred, and once patients were referred by HIV providers to speak with the team member overseeing consent and enrollment (Dr. Cabrera), we had very few rejections. As mentioned above, we did have patients who completed the survey and in-person clinical assessments but did not complete the DXA portion of the study prior to the closure of recruitment due to the pandemic, however, those patients were not included in the analysis. In terms of the chi-squared results mentioned in Table 2, we have double checked this analysis and updated it accordingly.

**Reviewer #2: Test between the time: if the distribution is not normal (which is likely), a non-parametric test should be used to compare the groups.**

*Response to Reviewer #2:* For variables that did not follow a normal distribution (for example, SPPB test and each of their categories) the Mann-Whitney U test were used. This has been updated under the Statistical Analysis paragraph starting in Line 166, page 6

**Reviewer #2: Separated analyses were performed for HIV-infected and uninfected participants and by outcomes. Yet, the two groups have different characteristics, thus the results may not be comparable unless some form of standardization is used.**

*Response to Reviewer #2:* Yes, the reviewer is correct that we did perform separate analysis for people living with and without HIV, and by outcomes, as were aiming to explore how HIV infection played a role on each outcome. We agree that the two groups have some different characteristics, however in the unadjusted regression analyses we systematically tested each independent variable shown in Table 1 and variables not shown in Table 2 were not selected for further testing in the multivariable models. They could have been removed due to multicollinearity or lack of association at the level required for consideration.

**Reviewer #2: Analyses were also performed separately by outcomes. Was sarcopenia associated with SPPB and/or grip strength?**

Yes, sarcopenia was associated with both SPPB and grip strength as the definitions utilized in this study for sarcopenia spectrum incorporated physical performance (measured by the SPPB) and physical strength (measured by grip strength). Therefore, we did not perform separate regression analyses to examine those associations. We have now clarified this in the Statistical Analysis section as follows: *“As body composition measures (and the SPPB and grip strength) are part of the EWGSOP algorithm for identifying sarcopenia spectrum, these were not included in the multivariable regression models for sarcopenia spectrum.”*

**Reviewer #2: DISCUSSION. To my view, the discussion needs additional work.
First, it seems to me that there is no significant difference in the prevalence of sarcopenia between the HIV-infected and uninfected groups (although the limited sample size is a problem). Therefore, the authors should be careful in their statement about greater prevalence of severe sarcopenia among HIV-infected women as it is not significant (only 4 and 2 cases, p = 0.8!). The same statement is repeated at the end of the discussion and in the abstract.**

*Response to Reviewer #2*: We appreciate your important comments regarding the Discussion section of our manuscript. We agree that it is important to make clear that there was no significant difference in sarcopenia prevalence between the two groups. We have revised the statement now in the Discussion to take the statement out of the first and last paragraphs of the Discussion, and we have modified the section where it is mentioned in Lines 382-386, page 20.

**Reviewer #2: I am not sure it is the first study to explore the relationship between body composition, sarcopenia, and physical function as it is claimed line 254.**

*Response to Reviewer #2*: We would like to clarify that the statement regarding the this being the first study in the Latin American and Caribbean region to study this topic using the rigorous DXA and SPPB assessment tools. We have now spelled out the acronym LAC throughout the manuscript to avoid misunderstanding or perceived misrepresentation. Prior to the start of this study, we performed a systematic review of studies examining aging-related co-morbidities among persons living with HIV in LAC, including sarcopenia. We found only one study from Brazil that assessed this outcome among 33 patients with HIV, and 60 patients without HIV, but this study used bioelectrical impedance to measure muscle mass, and gait speed to assess physical performance rather than more accurate DXA (BIA underestimates lean mass and overestimates fat mass when comparing individual level data), and comprehensive SPPB (which includes gait speed as one of three components to assess physical performance). To clarify our statement, we have now changed the words “well-established assessment tools” to *“using the rigorous DXA and SPPB assessment tools.”*

Reference: Cabrera DM , Diaz MM, Grimshaw, A, et al. Aging with HIV in Latin America and the Caribbean: a Systematic Review. Curr HIV/AIDS Rep

. 2021 Feb;18(1):1-47. doi: 10.1007/s11904-020-00538-7. Epub 2021 Jan 5. PMID: 33400168

**Reviewer #2: The authors found that women living with HIV have low physical performance, lower bone mass and fat mass, which is an important result that need to be further discussed. For instance, the authors compare their results with two studies conducted in the US (lines 288 to 303). Is this that there are no other studies with SPPB results among women? Otherwise, they should be more systematic. Studies conducted in other LMIC (especially LAC) would be more relevant (e.g. review by Bernard et al 2017 or Gomes-Neto et al, 2018).
I am also surprised to read that there is a scarce literature on the relation between SPPB and age (line 305) as SPPB was initially created to assess physical performance among older people.**

*Response to Reviewer #2:* We appreciate these comments, we agree about the importance of showing studies using the SPPB in Latin American and LMIC populations of PLWH in general. As mentioned above, our recent systematic review identified this as an important gap in the HIV literature for Latin America and the Caribbean. Along those lines, we also agree about the important findings reported by Gomes-Neto et al, 2018 and Bernard et al 2017 in terms of physical function decline and aging among HIV populations and have updated the Discussion section of the manuscript with those findings on line 316 and lines 347-351. We would like to clarify that our reference to scare literature was specific to the literature regarding PLWH and have now clarified this in line 335.

**Reviewer #2: Other studies have found that low bone density / OP to be more prevalent that sarcopenia among people living with HIV (e.g., Gregson et al. 2022). The authors may discuss further their results regarding bone density and compare with other studies.**

*Response to Reviewer #2:* We appreciate your comments for the Discussion. We agree regarding the importance of low bone mineral density and osteoporosis prevalence among persons aging with HIV. As mentioned above, this study was part of a larger study which had as its primary aim the measurement of vertebral fractures and osteoporosis in this population. That manuscript has recently been accepted for publication in *Archives of Osteoporosis*.

**Reviewer #2: The authors could give possible interpretation of the association between fat trunk and SPPB.**

*Response to Reviewer #2:* Regarding the trunk fat mass, as we mentioned on the manuscript, it was negatively associated with SPPB in the regression analysis for both women with and without HIV and these has been seen in previous studies in different age populations, however, we could not find any including people living with HIV. We agree with prior evidence suggesting that higher muscle fat infiltration can be an important predictor of physical performance and this relationship has been shown to be stronger in women than in men. This has been added on lines 339-343.

Reference: Shin et al. Relationship of Physical Performance with Body Composition and Bone Mineral Density in Individuals over 60 Years of Age: A Systematic Review. J Aging Res. 2011; 2011: 191896. Published online 2011 Jan 23. doi: 10.4061/2011/191896

**Reviewer #2: Physical activity is an important factor related to physical performance and body composition that could modify the association found. As it was not assessed, the authors should acknowledge and discuss this limitation. The authors should also acknowledge the multiple tests used and caution that the risk alpha is increased (in other words, some significant associations may just be significant by chance).**

*Response to Reviewer #2:* We also agree that the role of physical activity would be valuable to include in future analyses, and have added this to the Limitations. In terms of the concern for multiple tests, we did take care to streamline the number of variables included in each multivariable regression model by testing for potential multicollinearity between variables and setting a threshold for entry. However, we agree that it is always important to acknowledge the potential for finding associations by chance in the setting of multiple tests and have now mentioned this in the Limitations section.

**Reviewer #2: Minor comments:
Line 291: do the authors mean “uninfected”?
Line 266: the authors wrote that “Changes in body composition, such as fat redistribution, have been shown to impact physical function and risk for mortality [reference 1].” However, I am not sure that reference 1 addresses physical function? Additional references to support the statement need to be included.**

***Response to Reviewer #2:*** We appreciate your comments for the Discussion. We have updated the misspelled term “uninfected” in the indicated line. Regarding reference 1, this paper by Grant *et al.* measured early and late longitudinal body composition changes among people with HIV and discuss the implications of their findings on physical function and mortality, however we agree with the reviewer that they do not specifically measure these two outcomes directly in their study. Therefore to strengthen this statement we have now added two additional references to this section.
